# Study of the Peak to Background (P/B) Method Behavior as a Function of Take-Off Angle, Tilt Angle, Particle Size, and Beam Energy

**DOI:** 10.1155/2021/8070721

**Published:** 2021-10-08

**Authors:** Seyed Mahmoud Bayazid, Yu Yuan, Raynald Gauvin

**Affiliations:** ^1^Department of Mining and Materials Engineering, McGill University, Montreal, Quebec, Canada H3A 0C5; ^2^Object Research Systems, Montreal, Quebec, Canada H3C 1M4

## Abstract

Monte Carlo simulations were performed to investigate the behavior of the peak to background ratio (P/B) of particles on a substrate as a function of different variables such as take-off angle, tilt angle, particle size, and beam energy. The results showed that the P/B highly depends on the beam energy, the size of particles, and the composition of the substrates. Results showed that the rate of intensity reduction of the peak is less than the background for a high tilt angle (60 degrees), and thereby, the P/B increases at a high tilt angle. It was shown that by increasing the take-off angle, the P/B initially reduces and then reaches a plateau. Results showed that the P/B highly depends on the size of particles. Analyses showed that by moving the electron beam from the center to the side of the particle, the P/B increases. Finally, the spherical particles have higher sensitivity of the P/B to the beam position than the cubical particles.

## 1. Introduction

Converting the X-ray intensity into concentration for nonflat samples has been challenging for decades. Conventional methods such as ZAF or *φ*(*ρ*z) methods are valid for specimens with homogeneous composition and flat surfaces [1]. Some problems with the analysis of nonflat specimens are described in the following. First, the undefined incidence angle of electrons makes it difficult to model both the backscattered electrons and the depth distribution of the generated X-rays. Second, the take-off angle and the average depth of the generated X-rays are not well specified for nondefined geometries; thus, the absorption path length can change considerably. Moreover, some side-scattering might happen in the case of the particle's analysis due to the geometry factor [2]. Finally, for small particles, the randomization process of electrons has not been completed before they leave the particles. Therefore, the anisotropy in the bremsstrahlung X-ray may not be negligible [2–4]. Several methods including the Armstrong model [5], the peak to background (P/B) method [6, 7], and the Monte Carlo simulations [1] have been proposed to overcome this matter. Armstrong and Buseck [5] developed analytical approximations and calculated X-ray corrections iteratively in a similar manner to the ZAF method for the homogeneous composition. Armstrong's model is based on the classification of particles by shape. However, the computation of the multidimensional integrals required can be time-consuming [7]. Moreover, the dependency of the Armstrong model on the particle's shape limits its application in the characterization of nonflat surfaces [3]. On the other hand, in the case of rough surfaces, the P/B method [6–8] was proposed as a quantitative model, which is an extension of the Marshall-Hall model [9, 10] for the correction of mass loss in beam-sensitive materials. Declared by this method, the P/B is constant at any location on the rough surface, and that this ratio is the same as that of bulk material of the same composition having a flat surface. However, using Monte Carlo simulations, Gauvin and Lifshin [11] showed that the P/B is not constant for the rough surfaces. Gauvin demonstrated that the P/B has some weaknesses. For instance, the assumption that the P/B is independent of the specimen roughness is not strictly correct since the ionization cross-sections and the Bremsstrahlung cross-sections are not the same for rough surfaces. Rez and Konopka [3] indicated that the P/B increases with increasing overvoltage; therefore, the voltage dependence to the P/B ratio means that it is not truly independent of geometry. Researchers [4, 12] showed that the most likely source of error in the P/B method is the uncertainty in the calculation of the Bremsstrahlung spectrum intensity. However, Heckel and Jugelt [13] pointed that the influence of statistical errors of the Bremsstrahlung spectrum counts can be diminished by prolonging the measuring times. Some concerns in using the P/B for quantitative characterization of nonflat surfaces have still remained for decades. In this paper, the behavior of the P/B as a function of different parameters such as take-off angle, tilt angle, particle size, and beam energy are investigated using the Monte Carlo model to show “Does the efficiency of this method of analysis for particles or rough surfaces is enough?”

## 2. Materials and Methods

**Figure 1:**
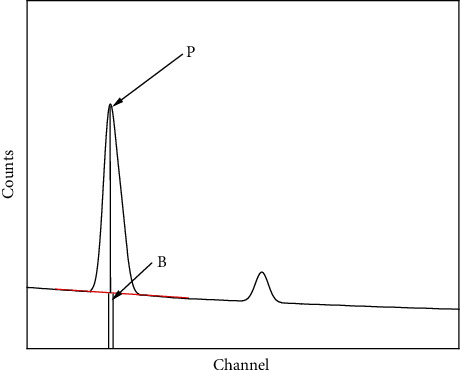
Definition of the P/B ratio. P is the net intensity of the characteristic line (e.g., K*α* line), and B is the background intensity at the line energy.

To calculate P/B ratios for different conditions, various Monte Carlo simulations were performed using the Monte Carlo program described in reference [14]. MC X-ray does X-ray microanalysis from the simulation of electron scattering in solids of different types of geometries. Scatterings are built based on an accidental process where electrons are simulated using a forward scattering random walk. The methodology, calculations, and physics of the program were described in reference [14]. The simulations were performed for spherical particles of Al deposited on substrates (C, Ti, Ag, and Au). In this work, the beam diameter was equal to 10 nm and 100000 electrons were used to simulate electron trajectories in order to compute X-ray emission for particles. The main variables used in these simulations were beam energy, tilt and take-off angles, particle size, particle's shape (sphere and cubic), and the composition of the substrate. The P/B was calculated for the total number of peak counts to the number of background counts under the peak (Figure 1).

## 3. Results

### 3.1. Beam Energy

**Figure 2:**
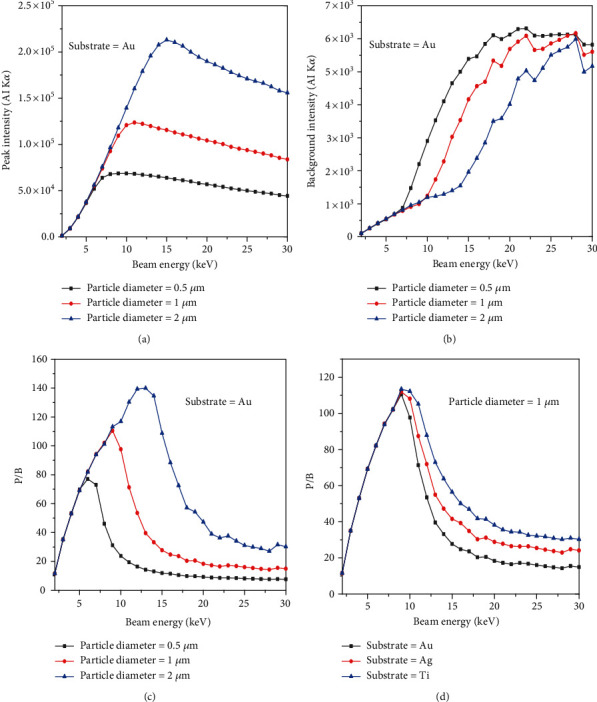
Variation of peak (Al-K*α* line) intensity, background (Bremsstrahlung X-ray) intensity, and the P/B as a function of beam energy.

Figure 2(a) shows the variation of the peak intensity for Al-K*α* as a function of beam energy when the beam is located at the center of spherical particles. The tilt and take-off angles were set at 0 and 40 degrees, respectively. The peak intensity increases with the beam energy, goes to a maximum value depending on the size of the particle, and then decays when the beam energy is further increased. Results show that the maximum value of the peak intensity depends on the size of the particle. The bigger the size of the particle, the higher the maximum value of the peak intensity. On the other hand, the intensity of the background (Bremsstrahlung X-ray) continuously increases as the beam energy increases. However, there is a small reduction in the background intensity when the beam energy is larger than 27 keV. The reason behind this phenomenon could be that by increasing the beam energy over 27 keV, most of the electrons pass through the particles rapidly (transfer to the substrate), depends on the size of particles, without generating more Bremsstrahlung X-rays in the particles. The background intensity is a function of the beam energy according to Kramers [15]. As the beam energy increases, the maximum continuum energy increases. Note that at certain beam energy (less than 5 keV), a small particle has higher background intensity in comparison with a big particle (Figure 2(b)) because of the electron transition phenomenon [4]. The peak and background intensities for all particles are the same as long as the beam energy is less than 5 keV.  Figure 2(c) indicates that not only the P/B changes with the size of the particle but also it varies with beam energy. Regardless of the particle size, the value of the P/B rises with beam energy, goes to a maximum, and then decays to a plateau. It also shows that the bigger the size of the particle, the higher the P/B value (for beam energies larger than 10 keV).  Figure 2(d) shows that the P/B depends on the substrate that the particle deposited on it. When the beam energy is more than 10 keV, the higher the atomic number of the substrate, the lower the P/B. For beam energies more than 10 keV, the electron range increases, and the X-rays are generated inside the substrate as well; therefore, this phenomenon could affect the P/B.

### 3.2. Tilt Angle

**Figure 3:**
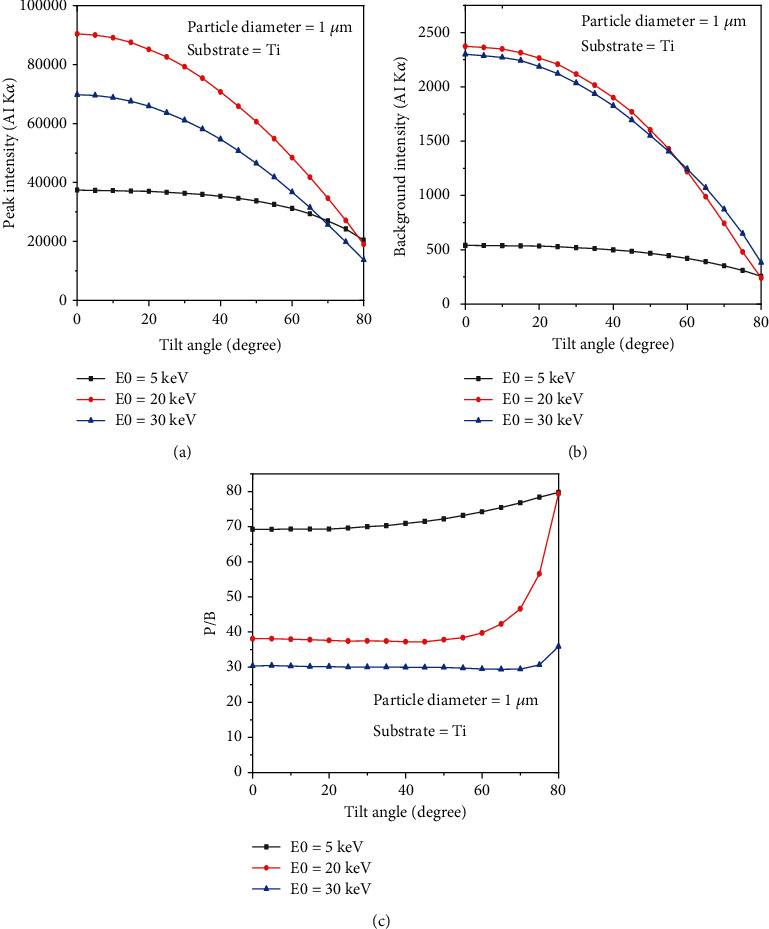
The peak intensity, background intensity, and the P/B as a function of tilt angle.

Although the peak intensity and background intensity decrease as the tilt angle increases, the P/B is almost stable from 0 to 60 degrees of tilting. It means that the rate decreasing for both intensities is nearly the same. However, the P/B increases as the tilt angle is larger than 60 degrees (see Figure 3). Results show that the P/B is roughly stable as a function of tilt angle when the beam energy is low (5 keV). On the other hand, for high beam energies (20 keV), the P/B grows from roughly 38 to 80 when the tilt angle changes from 60 to 80 degrees.

**Figure 4:**
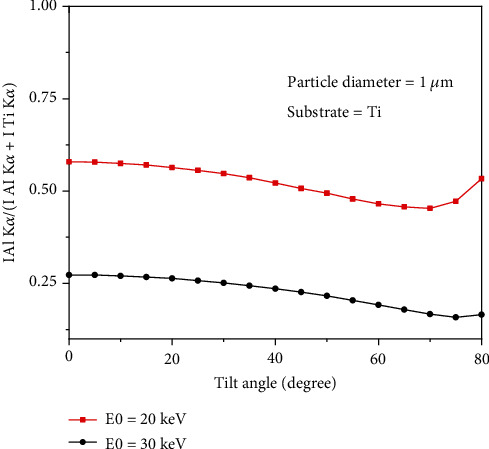
Variation of f ratio given by (I Al_K*α*)/(I Al_K*α*+I Au_M*α*) as function of tilt angle (Al deposited on Ti).

Figure 4 shows the variation of the *f* ratio [16] given by (I Al_K*α*)/(I Al_K*α*+I Au_M*α*) as a function of tilt angle. Results show that the *f* ratio decreases with increasing tilt angle for the beam energy of 20 keV, goes to the minimum value at 70 degrees, and then increases again by increasing the tilt angle. This trend is similar for the beam energy of 30 keV but the minimum value of the *f* ratio happens at 75 degrees.

### 3.3. Take-Off Angle

**Figure 5:**
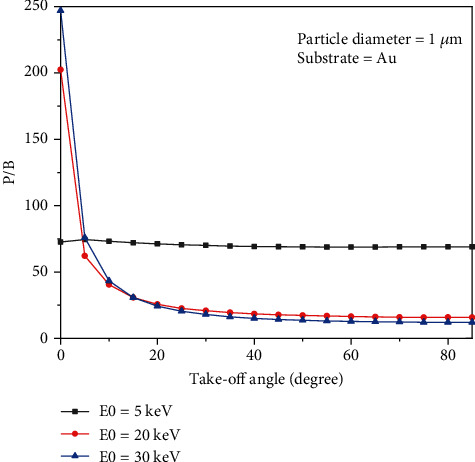
The P/B of Al-K*α* as a function of take-off angle.

**Figure 6:**
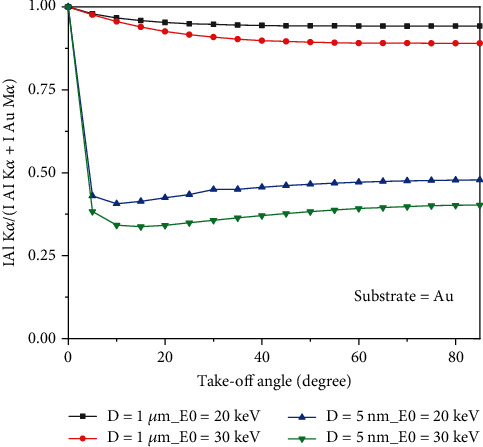
Variation of f ratio given by (I Al_K*α*)/(I Al_K*α*+I Au_M*α*) as function of take-off angle (Al particle deposited on Au).

Figure 5 shows the variation of the P/B for Al-K*α* as a function of the take-off angle. The beam diameter and tilt angle were 10 nm and 0 degrees, respectively. It is shown that the P/B does not change with the take-off angle at low beam energy (5 keV). However, for high beam energy values (20 and 30 keV), the P/B decreases as the take-off angle increases. Figure 5 shows that the P/B exponentially decreases when the take-off angle increases from 0 to 85 degrees. The variation of *f* ratio as a function of take-off angle is shown in Figure 6. Results show that the variation of the *f* ratio when the take-off angle increases is higher for small particles, for example, the variation of the *f* ratio when the take-off angle changes from 0 to 85 degrees for small particles (5 nm) is 51%; however, for a bigger particle (1 *μ*m), the *f* ratio variation is 10%. The *f* ratio for small particles (5 nm) decreases sharply by increasing the take-off angle, goes to a minimum, and then increases to a plateau. However, for particles with 1 *μ*m diameter, the *f* ratio decreases smoothly and continuously by increasing the take-off angle and reaches a plateau. Regardless of the particle size and the take-off angle, the higher the beam energy, the lower the *f* ratio.

### 3.4. Particle Size

**Figure 7:**
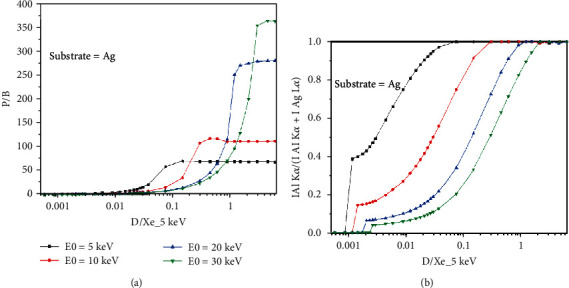
Variation of the P/B and f ratio as function of D/Xe (5 keV) for Al K*α*; Xe is the range of emitted X-rays for the bulk sample, in this case at 5 keV.

Figure 7(a) shows the variation of the P/B and f ratio as a function of particle size (D, particle diameter) normalized by X-ray emitted range (*X*_*e*_) at different beam energies. For a certain beam energy, when the size of the particle increases, the P/B increases monotonically and then goes to the plateau. The higher the beam energy, the plateau happens at a bigger particle diameter. For small particles (less than 500 nm), the higher the beam energy, the less the P/B due to the size of the interaction volume. The *f* ratio increases as the particle size increases (see Figure 7(b)). At any particle size, the lower the beam energy, the higher the *f* ratio.

**Figure 8:**
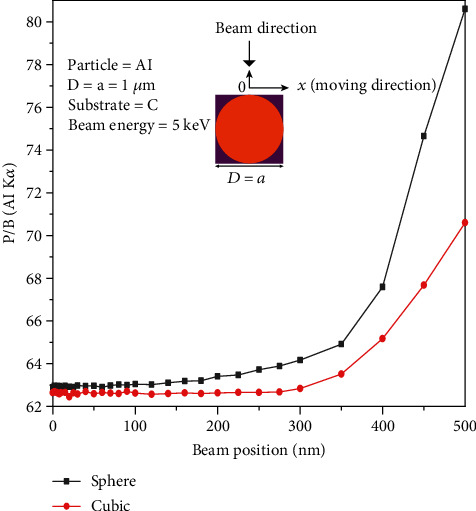
Variation of the P/B as a function of beam position.

Figure 8 shows that when the beam position is moved from the center of the particle (0) to the left side (the particle diameter = 1 *μ*m), the P/B is constant until 100 nm for both cubical and spherical particles. However, from this point (100 nm) all the way to the end, the P/B increases for both particles. Results show that the P/B for a spherical particle is more sensitive to the beam position than the cubical particle because the variation of the P/B in the case of spherical particle is more than cubical one. It could be concluded that the P/B depends on the particle shape and beam position.

## 4. Discussion

The presented results show that the P/B is not constant on a nonflat surface. Figures 2(c) and 2(d) indicate that not only the P/B highly depends on beam energy, but it relies on substrates for chemical characterization of particles. What is clear is that the P/B is less sensitive to the size of particles at low beam energy (less than 5 keV). It means that the size of particles at low beam energy (less than 5 keV) does not affect the P/B. However, when the beam energy is greater than 5 keV, the P/B is not the same for all particles (0.5, 1, and 2 *μ*m). This behavior can be explained via interaction volume. For low beam energies (less than 5 keV), the interaction volume is a part of particle volume (0.5, 1, and 2 *μ*m); however, when the beam energy increases, the interaction volume increases, and it can cover all over the small particles. Because the characteristic and Bremsstrahlung cross-sections [3] behave very similarly at high beam energies, thus the P/B tends to a constant at high beam energies. On the other hand, at high beam energies, the P/B changes when the substrate of the particle differs (Figure 2(d)), because at high beam energy, the interaction volume is too big and includes some part of the substrate. As a result, depending on the atomic number of the substrates, the backscattering affects the P/B value. Although it has been shown by Rez and Konopka [3] that the tilting does not affect the P/B in the range of 0 to 40 degrees, our results showed (Figure 3(c)) that the P/B changes for high tilt angles (60 to 80 degrees), especially at high beam energies. At high tilt angles (60 to 80 degrees), the number of electrons that penetrate to the particle to generate X-rays reduces; thus, the peak and the background intensities decrease as well. But as Figures 3(a) and 3(b) show, the rate of intensity reduction of the peak is less than the background; therefore, the P/B increases at a high tilt angle. The variation of the *f* ratio with tilt angle (see Figure 4) is in agreement with the proposed assumption. Figure 5 shows that the take-off angle has a high impact on the P/B, especially at high beam energies, since the absorption path increases (if the particle size and the X-ray position remain the same) as the take-off angle goes from 0 to 40 degrees for a spherical particle. However, at low beam energy (5 keV), the take-off angle does not affect the P/B, because the interaction volume is very small in comparison with the situation when the beam energy is 30 keV. Therefore, the absorption path is almost constant as the take-off angle changes. Moreover, increasing the take-off angle more than 40 degrees does not change the P/B. It could be explained that when the take-off angle is more than 40 degrees, the number of emitted X-rays that could reach to the detector is very small and the geometry does not have big effect on the absorption path. Figure 7(a) shows that the P/B is affected by the particle size. Although Statham and Pawley [7] claimed that the P/B is the same for particles with diameter 3 *μ*m and 9 *μ*m at beam energy of 20 keV. Nevertheless, our results showed that the P/B is not constant when the size of particles is changed between 1 and 5 *μ*m at beam energy of 20 keV. At the beam energy of 5 keV, the P/B changes when the particle diameter goes from 1 to 500 nm. However, the P/B does not change for particles larger than 500 nm. The problem with the quantitative microanalysis of particles is that electrons can be scattered from all sides of particles, so the generated X-rays depend on the size and shape of particles [7, 17]. On one hand, the characteristic X-ray generated within the particle is only a fraction of the X-ray generated in a bulk sample of the same composition, when particle sizes are below the interaction volume of the electrons [3]. On the other hand, the shape of a particle makes it very difficult to correctly consider the path of characteristic X-rays between their generation locations and the particle surface [7, 17]. Therefore, geometric factors impact the measured X-ray intensities and thereby the P/B and the quantitative analysis of particles [3, 17, 18]. Another reason that shows the P/B highly depends on the geometry and the beam position is shown in Figure 8. Results showed that by moving from the center to the side of the particle, the P/B increases, and this P/B boosting is more for the spherical particle than the cubical one. Therefore, it could be concluded that the P/B pertains to the geometry and the beam position. Similarly, Newbury [17] has reported that the intensity of a peak varies if the beam position is changed on the particle.

## 5. Conclusion

The peak to background ratio can be affected by many factors. In this study, the P/B was analyzed while beam energy, tilt angle, take-off angle, and particle size were changing. It was shown that not only the P/B highly depends on beam energy and the size of particles, but it relies on substrates. Results showed that at a high tilt angle, the rate of intensity reduction of the peak is less than the background; therefore, the P/B increases. Moreover, the tilting cannot affect the P/B at the range of 0 to 40 degrees. On the other hand, the take-off angle highly affects the P/B of the particles. The higher the take-off angle, the lower the P/B. The dependency of the P/B to the take-off angle increases when the beam energy increases. The effect of particle size at different beam energies on the P/B showed that the P/B is not constant when the size of particles is changed, depending on the beam energy. More investigation showed that by moving from the center to the side of the particle, the P/B increases; this P/B enhancing is more for the spherical particle than the cubical one. It could be concluded that the P/B depends on the geometry and the beam position.

## Data Availability

The data that support the findings of this study are available from the corresponding author (Seyed Mahmoud Bayazid, email: mahmoud.bayazid@mail.mcgill.ca) upon reasonable request.
